# *In vitro* Endothelialization and Platelet Adhesion on Titaniferous Upgraded Polyether and Polycarbonate Polyurethanes

**DOI:** 10.3390/ma7020623

**Published:** 2014-01-24

**Authors:** Karla Lehle, Jing Li, Hanngörg Zimmermann, Björn Hartmann, Daniel Wehner, Thomas Schmid, Christof Schmid

**Affiliations:** 1 Department of Cardiothoracic Surgery, University Medical Centre Regensburg, Franz-Josef-Strauss-Allee 11, 93042 Regensburg, Germany; E-Mails: jing.li@ukr.de (J.L.), christof.schmid@ukr.de (C.S.); 2 PFM medical titanium, Höfener Str. 45, 90431 Nürnberg, Germany; E-Mails: hanngoerg.zimmermann@pfmmedical.com (H.Z.); bjoern.hartmann@pfmmedical.com (B.H.); 3 Dualis Medtech GmbH, Am Technologiepark 8+10, 82229 Seefeld, Germany; E-Mails: daniel.wehner@dualis-medtech.de (D.W.); thomas.schmid@hotmail.de (T.S.)

**Keywords:** endothelial cell seeding, cytocompatibility, haemocompatibility, platelet adhesion, cardiovascular tissue engineering, titanium

## Abstract

Polycarbonateurethanes (PCU) and polyetherurethanes (PEU) are used for medical devices, however their bio- and haemocompatibility is limited. In this study, the effect of titaniferous upgrading of different polyurethanes on the bio- and haemocompatibility was investigated by endothelial cell (EC) adhesion/proliferation and platelet adhesion (scanning electron microscopy), respectively. There was no EC adhesion/proliferation and only minor platelet adhesion on upgraded and pure PCU (Desmopan). PEUs (Texin 985, Tecothane 1085, Elastollan 1180A) differed in their cyto- and haemocompatibility. While EC adhesion depended on the type of PEU, any proliferative activity was inhibited. Additional titaniferous upgrading of PEU induced EC proliferation and increased metabolic activity. However, adherent ECs were significantly activated. While Texin was highly thrombotic, only small amounts of platelets adhered onto Tecothane and Elastollan. Additional titaniferous upgrading reduced thrombogenicity of Texin, preserved haemocompatibility of Elastollan, and increased platelet activation/aggregation on Tecothane. In conclusion, none of the PUs was cytocompatible; only titaniferous upgrading allowed EC proliferation and metabolism on PEUs. Haemocompatibility depended on the type of PU.

## 1. Introduction

Polyetherurethanes (PEU) and polycarbonaturethanes (PCU) are used in implantable medical devices because of their relatively superior biocompatibility and attractive mechanical properties (high elongation capacity, good abrasion resistance, high flexibility, and good biocompatibility for short-term usage) [1–5]. However, long-term contact-time of blood with artificial surfaces of medical devices such as small-caliber vascular grafts, heart valves, cardiac assist devices, and total artificial hearts increase the risk of thromboembolic complications that may lead to treatment failure. Endothelialization of the blood-contacting surfaces of medical devices would contribute to an antithrombogenic and anti-inflammatory coating that mimics the native lining of blood vessels and the heart [6]. Full coverage of polymer surfaces with endothelial cells (ECs) prevents platelet adhesion, thrombus deposition, inflammatory cell infiltration, and neointimal formation [7]. Recent studies have shown that rapid re-endothelialization or *in vivo* endothelialization with precursor cells on the surface of devices provides an inherent nonthrombogenic potential [8,9]. Thus, the function of endothelialization and thromboresistance of medical device (such as cardiovascular devices) surfaces has been regarded as important means to improve their respective treatment effect.

Various strategies in cardiovascular tissue engineering have been used to improve endothelialization of polymer surfaces [10]. A recent approach to improve biocompatibility of polymers for biomedical applications is titanium (Ti) upgrading [11–14]. Thin films of titaniumcarboxonitride on different polymer surfaces maintained flexibility of the polymer, increased wettability of the surfaces and supported EC adhesion and sustained anti-inflammatory function of the cells [12,15].

In this study, plasma-activated chemical vapour deposition (PACVD) technique was used to upgrade different PEUs and PCUs with a Ti-based layer [16]. The cyto- and haemocompatibility of titaniferous upgraded PUs were tested under static culture conditions.

## 2. Results

### 2.1. XPS of Polyurethanes after Titaniumcarboxonitride Upgrading

**Figure 1.**
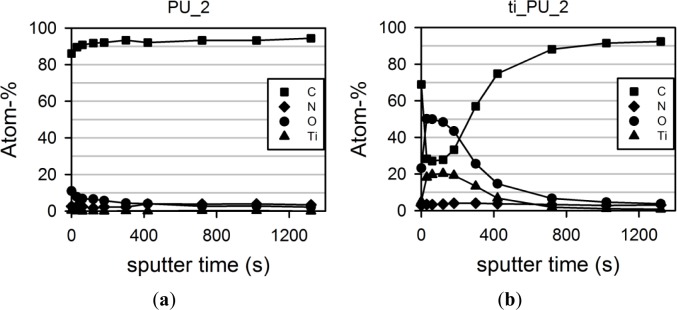
Representative XPS of (**a**) pure and (**b**) titaniferous upgraded PU_2. Surface characteristics show a homogenous composition of carbon (C), oxygen (O), titanium (Ti) and nitrogen (N) on titaniferous upgraded ti_PU_2 and pure PU_2 (control). The spectrum of the upgraded surface which can be regarded lies in the measuring range between 30 and 1000 sputtering seconds.

The maximal surface atom fraction of titanium (Ti) was between 19% and 21%, which decreased toward the inner part of the polyurethane (with increasing sputter time). Figure 1 presents the curve progression of pure (Figure 1a) and titaniferous upgraded PU_2 (Figure 1b). XPS analysis of titaniferous upgrading of the other polyurethanes provided similar curve progressions. The concentration of carbon atoms (C), the main component of polyurethane, increased with penetration depth. The upgraded surface was composed of carbon (C), titanium (Ti) and nitrogen (N), each being a component of the upgrading reagent Tetrakis (dimethylamino)titanium (Ti[N(CH_3_)_2_]_4_). When exposed to air during storage, the upgraded surface absorbs oxygen (O). During plasma deposition, no oxygen was present.

### 2.2. Cytocompatibility of Pure and Titaniferous Upgrading PUs Using EC Adhesion

**Figure 2.**
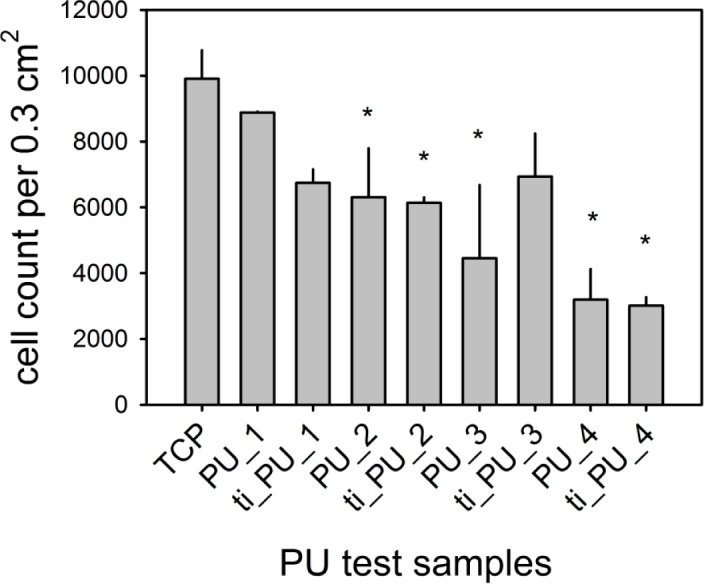
An *in vitro* study (*n* = 4) over 24 hours about adhesion of human endothelial cells seeded onto different PUs. Cells were seeded (20,000 cells per 0.3 cm^2^) onto test samples made of polyetherurethanes (PEU) (PU_1, PU_2, PU_3) and polycarbonateurethanes (PCU) (PU_4) without and with titaniferous upgrading (ti_PU) and tissue-cultured polystyrole (TCP) for 24 hours. Data are presented as mean with standard deviation. Statistical analysis included comparison of test materials with TCP (*, *p* ≤ 0.05).

As shown in Figure 2, adherence of ECs depended on the type of PU. While ECs avoided cell adhesion onto polycarbonate-based PU (PU_4), the cells preferred adherence onto polyether-based PUs. Highest cell density was documented for PU_1 (90% ± 1% of TCP), followed by PU_2 (64% ± 15% of TCP; *, *p* ≤ 0.05) and PU_3 (45% ± 22% of TCP; *, *p* ≤ 0.05). Only titaniferous upgrading of PU_3 tended to improve EC adhesion (not significant).

**Figure 3.**
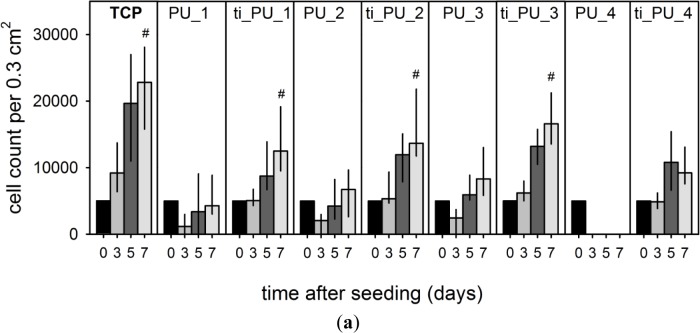

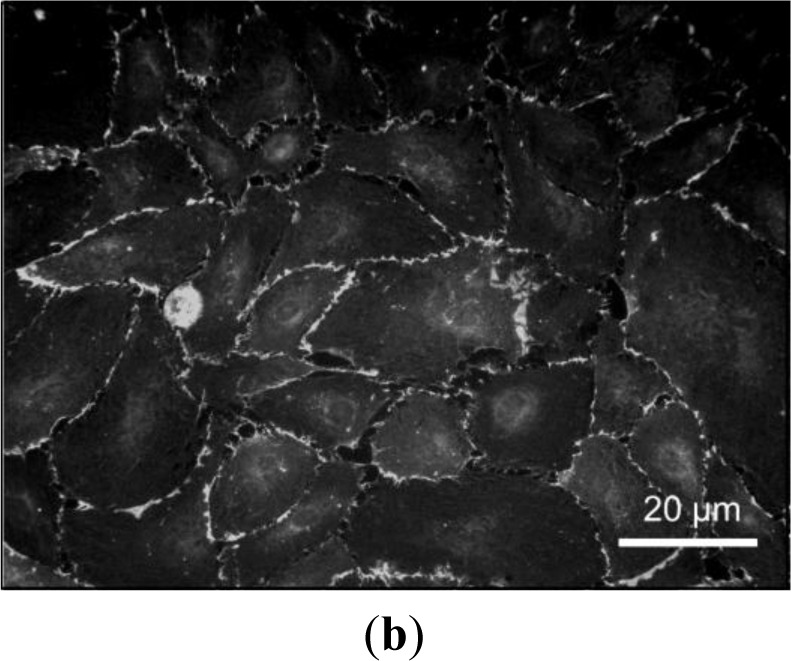
An *in vitro* study (*n* = 16) over seven days regarding proliferation of human endothelial cells seeded onto different PUs. (**a**) Cells were seeded (5000 cells per 0.3 cm^2^) onto test samples made of PEU (PU_1, PU_2, PU_3) and PCU (PU_4) without and with titaniferous upgrading (ti_PU) and tissue-cultured polystyrole (TCP). Cell counts were analyzed on day 3, 5 and 7 after seeding. Data are presented as median (25/75th percentile). Statistical analysis included increase in cell count from day 3–7 (#, *p* ≤ 0.001). Cell count on day 7 was highest for TCP and significantly lower for ti_PU_1 (*p* = 0.002), ti_PU_2 (*p* = 0.015), ti_PU_3 (*p* = 0.056); (**b**) on day 7 after seeding cells formed a confluent monolayer onto PEUs. One representative sample presented CD31-FITC stained EC.

Cell proliferation was an essential criterion for cytocompatibility of different biomaterials (Figure 3). While the count of ECs cultured on TCP increased significantly from day 3 to 7 after seeding (by a factor of 2.4 ± 0.7, *p* ≤ 0.001), cell density remained unchanged over the whole study period for cells seeded onto all pure PUs. Additional titaniferous upgrading initiated cell proliferation but to a lesser extent than for cells on TCP. No cell proliferation was observed for cells seeded onto titaniferous upgraded polycarbonate-based PU (PU_4). Seven days after seeding, cell number on titaniferous upgraded polyether-based PUs was significantly higher than on pure PUs (each test sample, #, *p* ≤ 0.001). However, maximum cell density was significantly lower than for cells cultured onto TCP (ti_PU_1, factor 1.7 ± 0.7, *p* = 0.002; ti_PU_2, factor 1.2 ± 0.4, *p* = 0.015; ti_PU_3, factor 1.3 ± 0.3, *p* = 0.056) (Figure 3a). Nevertheless, ECs cultivated for seven days on PEUs formed a confluent monolayer (Figure 3b). Similar data were documented using a cell viability assay (MTS-assay). On day 7 after seeding, the MTS absorbance of cells cultured onto titaniferous upgraded PEUs tended to be higher than for cells on pure PEUs (ti_PU_1 *vs.* PU_1, 0.15 ± 0.03 *vs.* 0.14 ± 0.11, not significant; ti_PU_2 *vs.* PU_2, 0.20 ± 0.09 *vs.* 0.08 ± 0.05, p=0.091; ti_PU_3 *vs.* PU_3, 0.15 ± 0.10 *vs.* 0.0 ± 0.05, not significant).

**Figure 4.**
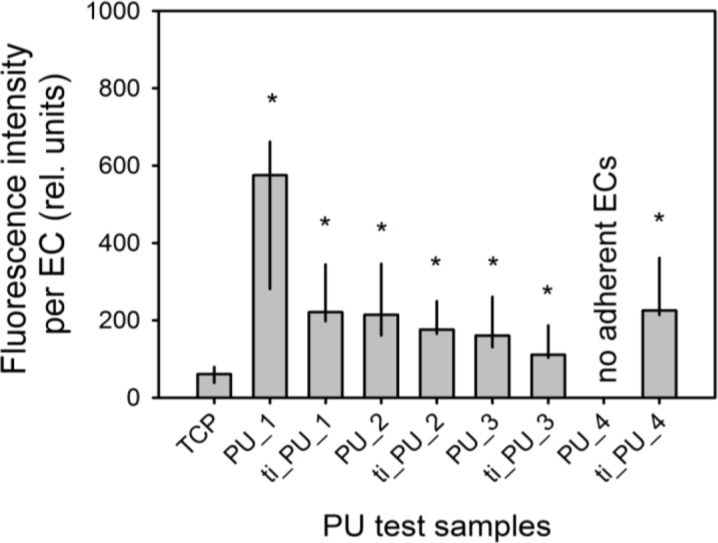
Activation of endothelial cells (EC) cultured on different PUs. ECs (*n* = 8) were cultured for 7 d (Figure 2), and incubated with Calcein-AM-stained PBMCs as described in Materials and Methods. The fluorescence intensity describes the amount of PBMCs that adhered on the surface of a single EC. Data are presented as median (25/75th percentile). The fluorescence intensity per EC increased significantly for all cells cultured onto different PU test samples (*, *p* ≤ 0.05).

Cultivation of ECs on the surface of different pure and titaniferous upgraded PUs could initiate an inflammatory activation of ECs. After seven days, a low proportion of PBMCs adhered onto the surface of ECs cultured on TCP. Fluorescence intensity increased significantly with respect to PBMC adhesion onto ECs cultured on different PUs. Assuming that PBMCs adhered only on activated ECs and considering differences of EC density on day 7 after seeding (Figure 3), fluorescence intensity per EC increased significantly for all cells cultured onto different PU test samples (Figure 4).

### 2.3. Haemocompatibility of Pure and Titaniferous Upgraded PUs Using Platelet Adhesion Test

**Figure 5.**
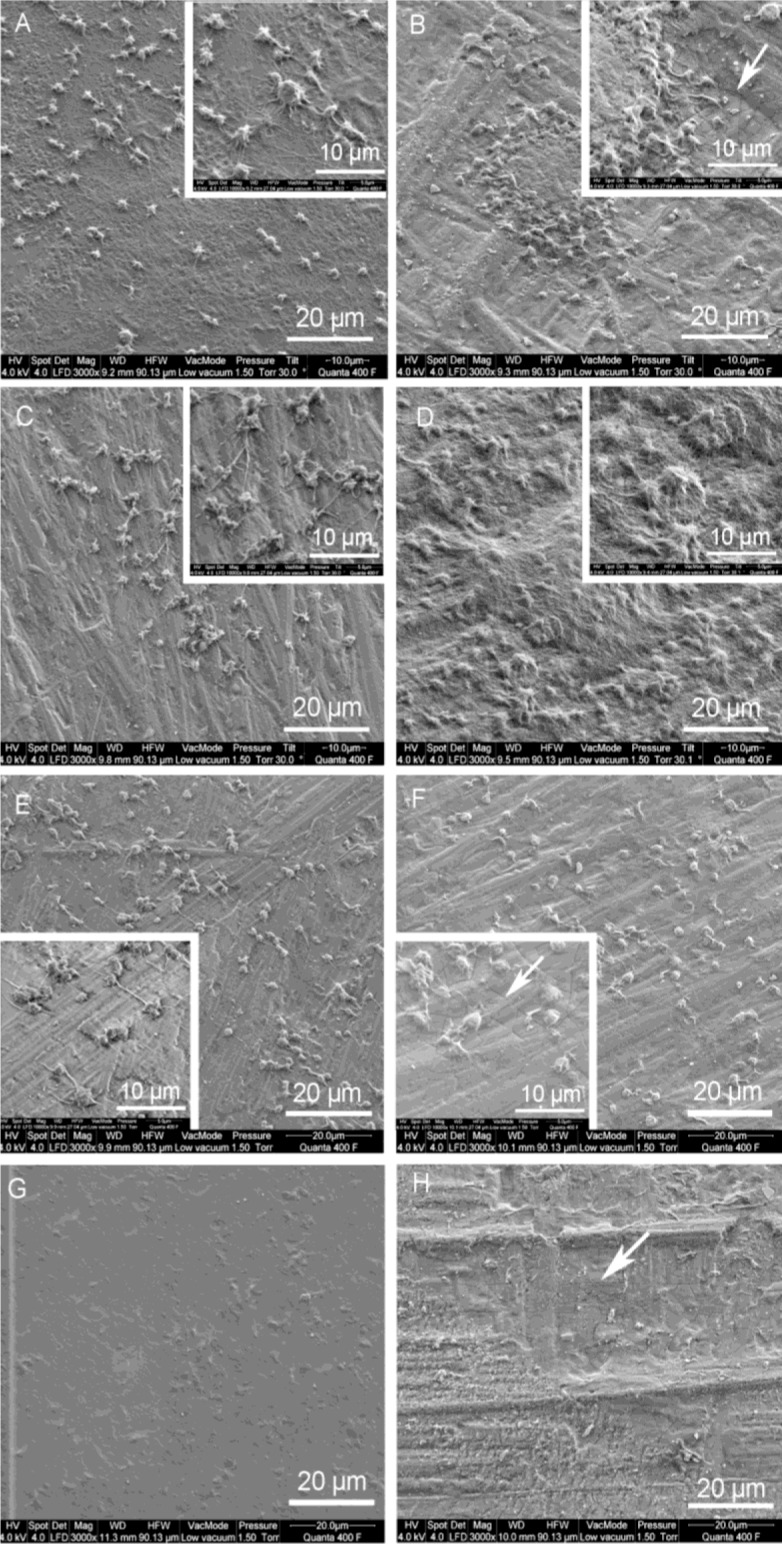
Morphologies of adherent platelets on the surface of different PUs. Isolated platelets were incubated with pure and titaniferous upgrading PUs. Representative SEM photographs of adherent platelets (3000× magnification) on (**A**) PU_1; (**B**) ti_PU_1; (**C**) PU_2; (**D**) ti_PU_2; (**E**) PU_3; (**F**) ti_PU_3 presented different cell density on the surface of the test samples; (**G**) PU_4 and (**H**) ti_PU_4 were more or less free of platelets. Arrows indicate small cracks in the titaniferous layers.

Representative SEM photographs of platelet adhesion onto PU test samples were shown in Figure 5. There was no platelet adhesion onto pure and titaniferous upgraded polycarbonate-based (PU_4) PUs (Figure 5G,H). Pure PEUs differed in their haemocompatibility. While adherent platelets onto PU_1 formed a dense network of collagen, PU_2 and PU_3 allowed minor platelet adhesion with single cells and small aggregates (Table 1).

Adherent platelets showed a dendritic shape with pseudopodia of varying lengths (tended to be longer on PU_2). Titaniferous upgrading of PU_1 reduced extensive platelet adhesion. Nevertheless, small clusters of aggregated platelets were detected that indicate the potential risk of platelet adhesion/aggregation on this surface. Titaniferous upgrading of PU_2 caused extensive platelet aggregation and network formation. In contrast, titaniferous upgrading of PU_3 did not affect the platelet adhesion. Cell density and shape was similar to pure PU_3 (Table 1, Figure 5). Finally, pure and titaniferous upgraded PU (PU_4) was thromboresistant; only few platelets adhered onto the surface, characterized by small and round cell morphology (Table 1, Figure 5).

## 3. Discussion

Titaniferous upgrading using PACVD technique facilitated proliferation of endothelial cells onto PEUs but not PCUs. Titaniferous upgrading of Texin^®^ 985 reduced platelet adhesion.

The great importance of EC coverage of synthetic surfaces in tissue engineering has been shown *in vitro* [18–21] as well as in animal studies [9,22]. One way to improve endothelialization of cell-repellent surfaces such as PU was nanoupgrading with titaniumcarboxonitride using PACVD technology [12,23]. In a previous study, we demonstrated poor adhesion and proliferation of human ECs and mouse fibroblasts (L929) on different types of pure PEUs [23]. Now, we could show that titaniferous upgrading initiated EC proliferation and increased metabolic activity to form a confluent monolayer on the surface of different types of PEU. The improved proliferative activity of ECs based on the increased wettability of titaniferous upgraded PEUs as already shown in an earlier study [12]. However, the effectiveness of endothelialization was limited compared to TCP. One reason might be the presence of small cracks in the titanium layer as presented in high-resolution SEM figures (Figure 5, inserts). These cracks were free of titanium (data not shown) and presented the pure PEUs to the ECs. In contrast, neither upgraded nor pure PCU allowed EC proliferation. Therefore, we suspect that EC seeding onto this type of PCU is inappropriate for enhancing biocompatibility of medical devices. The beneficial properties of PCU—the reported resistance to oxidative biodegradation [24–26]—for long-term biomedical usage could thus not be exploited. Only biofunctionalization of PCUs with the bioactive RGD peptide, which is a functional domain of fibronectin, allowed relatively rapid endothelialization with endothelial progenitor cells in an animal model [27]. Therefore, additional surface modifications of PCU are necessary to improve the potential of *in situ* endothelialization cardiovascular prostheses.

In addition to the increased proliferative activity of human ECs being in direct contact with titaniferous upgraded PEUs, the surface properties of upgraded PEUs initiated an activation of adherent ECs. The use of a functional test—the adhesion of PBMCs—indicated the presence of activated ECs on the surface of pure and upgraded PEUs. Independent of the type of PU and upgrading process, all adherent ECs bound significantly more PBMCs than ECs on the reference material (TCP). This was in contrast to our previous *in vitro* study [12]. Nanoupgrading with titaniumcarboxonitride of different polymer materials (PU, silicon, polypropylene, polytetrafluoroethylene, polyethylene terephthalate) did not affect proinflammatory response of human ECs [12]. Inconsistent results might be due to the large variability of PU materials showing different surface properties [3]. Of particular importance is the surface energy of PUs (controlled by the chain structures of PU, polar interactions and hydrogen bonds between their soft and hard segments), which affects its hydrophobicity [28]. The relevance of EC activation after contact with different biomaterials was already shown in experimental studies. Cenni *et al.* [29] reported an upregulation of E-selectin by human umbilical vein ECs (HUVEC) cultured on knitted Dacron in contrast to HUVECs cultured on pyrolytic carbon-coated polyethylene terephthalate and woven Dacron. Furthermore, Margiotta *et al.* [30] presented an increased expression of ICAM-1 by ECs grown on Dacron and expanded Teflon. In summary, each surface modification of polymers required individual bio- and cytocompatibility testing to exclude biomaterial induced inflammatory activation of ECs that might affect the success of cardiovascular devices.

Because thromboembolic formation is initiated by platelet adhesion to foreign materials [31], we investigated whether titaniferous upgraded PUs would decrease platelet adhesion. The type of PCU used in the present study prevented platelet adhesion. Titaniferous upgrading did not affect its anti-thrombotic properties. A polycarbonate-based PU (not further characterized) used by Weisenberg and Mooradian [32] demonstrated platelet adhesion consisting of round shaped and less spreading platelets—a marker of non-activated platelets. They concluded that silicon-based biomaterials may be more reactive to platelets and therefore more thrombogenic than PCU [32]. In another *in vitro* study, specified PCUs—Carbothane, Chronoflex, Corethane 80A and Corethane 55D—demonstrated some level of hemolysis after direct contact with blood for a 2 h period [33]. However, platelet adhesion in an *ex vivo* circuit was only significantly increased for Carbothane. In the same study, the PEUs (Tecoflex, Tecothane) recorded a low level of platelet adhesion. In our study, there were variations in the degree of platelet adherence on the surface of pure PEUs: While Texin (PU_1) was completely covered with a collagen network of aggregated and activated platelets, Tecothane (PU_2) presented a lower density of adherent platelets. However, the dendritic shape of adherent platelets with long pseudopodia indicated higher activation status of the platelets compared to Elastollan (PU_3). Titaniferous upgrading reduced platelet adhesion onto Texin (PU_1). Nanoupgrading with titanium using PACVD technique was already used in animal models to demonstrate its improved haemocompatibility. Implantation of titaniferous upgraded pump chambers made of PTFE from biomechanical hearts [34] in goats reduced thrombogenicity of the synthetic surface. Only eight weeks after implantation, a thrombus was formed on the luminal surface of the pure pump chambers resulting in a decrease of the pumping capacity. In contrast, titaniferous upgrading of the pump chambers prolonged their lifespan without detection of thrombotic depositions (Guldner, personal notification). Furthermore, titanium upgrading of clinically approved cardiovascular patches made of expanded polytetrafluoroethylene (ePTFE) enhanced the retention of umbilical cord tissue-derived mesenchymal stem cells, thus offering a potential cell reserve for repair of the damaged myocardium [35]. Finally, titanium nanoupgrading on glutaraldehyde-fixed bovine pericardium prevented immunorejection and created the first self-seeded glutaraldehyde-fixed biological heart valve within the circulation under arterial pressure [36,37].

The increased thrombogenicity of Tecothane (PU_2) after titaniferous upgrading was surprising. The causal relationship for this finding is unknown. It could be speculated that titaniferous upgrading of Tecothane (PU_2) affected the surface characteristics dramatically and favoured platelet adhesion. In contrast, coverage of the surface of Texin (PU_1) with the titaniumcarboxonitride-layer increased its thromboresistance. Nevertheless, small clusters of aggregated platelets were detected that indicated the potential risk of platelet adhesion/aggregation on this surface. Finally, only the direct blood contact in an animal model might bring light into the dark of improvement of haemocompatibility after titaniferous upgrading.

## 4. Experimental Section

### 4.1. Test Samples and Titanium Upgrading

Discs (area, 0.3 cm^2^) of different types of polyether- (PU_1–3) and polycarbonate-based (PU_4) polyurethane foils (Table 2) were upgraded with a thin (50–80 nm) titaniumcarboxonitride layer (tiPU) by plasma-activated chemical vapour deposition(PACVD) (pfm medical titanium, Nürnberg, Germany) [12]. This upgrading technology (patent number EP 0 897 997 B1) used a precursor (Tetrakisdimethylamidotitanium, Ti(N(CH_3_)_2_)_4_) that is transferred into the gas phase and brought into the reactor by a carrier gas, such as hydrogen gas, under vacuum conditions. The precursor reacts with the substrate creating a resistant layer. Physical plasma is able to supply the substrate with high energy while the temperature during deposition remains low (approximately 30–35 °C). Within nonthermal plasma with high electron temperatures but with neutrons and ions at room temperature, only electrons can follow a quickly changing electrical field with a typical frequency of 13.56 MHz [16,38]. Thickness of the titaniferous layer depends on the plasma activation time. The elements and their composition of the titaniferous upgraded polyurethane (ti_PU) membranes were analyzed by XPS (X-ray Photoelectron Spectroscopy; Sage 100, SPECS, Berlin, Germany). Pure and upgraded test samples were washed with ethanol (70%) and sterile isotonic phosphate buffer saline (PBS), placed on the bottom of wells from a 96-well microplate (Nunc^®^, Wiesbaden, Germany), and fixed with sterile steel rings. Tissue-cultured polystyrol (TCP) (Nunc^®^) was used as a reference.

### 4.2. EC Adhesion and Proliferation—A Measure of Cyto- and Biocompatibility

Isolation and cultivation of human saphenous vein EC (HSVEC) was described in detail earlier [12]. Informed consent was obtained from cell donors and the protocol was approved by the local human ethics committee (No. 99/133). EC were cultured in growth medium with serum (GMS, Medium 199, 10% fetal calf serum, L-Glutamine (PAA Laboratories, Pasching, Austria), Supplement Pack (PromoCell, Heidelberg, Germany)) at 37 °C under 5% CO_2_. For EC adhesion tests, EC were seeded at a density of 66,000 cells per cm^2^ onto fibronectin-coated (10 μg/ml; Merck, Darmstadt, Germany) test samples or TCP and incubated for 24 h. Fibronectin-coating secured improved cell adhesion [15]. For EC proliferation tests, 17,000 HSVEC per cm^2^ were seeded onto fibronectin-coated test samples for 7 d. Cells were fed with fresh GMS on day 3 and 5 after seeding, harvested on days 3, 5 and 7 by addition of trypsin/EDTA (Promocell), and counted with an automatic cell counter (CASY-TTC, Roche, Mannheim, Germany) that allowed differentiation between live and dead cells. Furthermore, cell viability of EC was measured on day 7 using the MTS [3-(4,5-dimethylthiazol-2-yl)-2,5-diphenyl-tetrazoliumbromide] test (Promega, Madison, I) according to manufacturers’ instructions. In a subset of experiments, EC were stained with a mouse-monoclonal anti-human CD31-FITC (fluorescein isothiocyanate) antibody (DAKO, Glostrup, Denmark) and visualized with a fluorescence microscope (Leica DMRBE, Biberach, Germany).

### 4.3. Platelet Adhesion—A Measure of Haemocompatibility

Scanning electron microscopy (SEM) was used to measure platelet adhesion and to visualize shape changes of adherent platelets as a marker of activation. Briefly, human citrated venous blood was drawn from healthy male volunteers with written consent in accordance with the institution’s ethical guidelines (No. 10-101-0159). Blood samples were centrifuged (300 g, 15 min, 37 °C). Platelet-rich plasma (PRP) was isolated from the interphase, transferred into a polypropylene syringe containing 10% aqueous citrate dextrose (80 mg citrate, 120 mg glucose monohydrate in 10 mL H_2_O), and centrifuged again to discharge serum (PPP, platelet poor plasma). Platelets were counted using a Neubauer Haemocytometer. PRP (50 μL; 5 × 10^7^ platelets per 0.3 cm^2^) was coincubated with test samples (30 min, 37 °C, 5% CO_2_). After rinsing, the specimen were fixated in 2.5% glutaraldehyde in 0.1 M cacodylate buffer at 4 °C; post fixation was performed in 1% osmium tetroxide followed by critical point drying. After drying, the specimens were mounted on aluminum stubs (Ted Pella, Redding, CA, USA) with carbon disks (Ted Pella) and colloidal quick drying silver paint (Ted Pella). Then, the specimens were coated with gold-palladium, by means of a sputter-coater (E-5100, Polaron Equipment, Watford, England) at 10 mA for 1 min. The PU test samples were observed under an environmental, variable pressure Field Emission Scanning Electron Microscope (FESEM) (FEI Quants 400FEG, Philips, Eindhoven, The Netherlands) at an accelerating voltage of 5 kV and a working distance of 12–13 mm. The morphologies of adhered platelets were characterized by and classified according to platelet shape [17,39] (1, round or discoid, no pseudopodia present; 2, dendritic, early pseudopodial, no flattening; 3, spread dendritic, more pseudopodia flattened, partial hyaloplasm between pseudopodia; 4, fully spread, hyaloplasm fully spread, no distinct pseudopodia, formation of collagen networks).

Before the platelet adhesion tests were performed, the activation status of all PRP samples were tested using flow cytometry. A small proportion of PRP was double-stained with fluorochrome-conjugated monoclonal anti-CD41-FITC and anti-human CD62P-APC (BD Biosciences, Erembodegem, Belgium) (24 h at 4 °C) and analyzed. Only PRP preparations with less than 5%–10% CD41+CD62P+ platelets were used for platelet adhesion tests.

### 4.4. Adhesion of PBMC onto ECs Cultured on Different PUs

Fresh venous blood from healthy adult volunteers was collected in EDTA tubes. Peripheral blood mononuclear cells (PBMC) were isolated using Ficoll-Paque™ (1.073 g/mL; GE Healthcare, Uppsala, Sweden) [40]. PBMCs were stained with calcein-acetomethylester (calcein-AM; stock solution: 1 mg/mL in DMSO; working concentration: 5 μg/mL; Molecular probes, Eugene, OR, USA), counted (Neubauer Haemocytometer), resupended in GMS (2 × 10^5^ PBMCs per well), and colocalized with endothelialized test samples for 30 min. Test samples were washed with phosphate buffer saline (PBS), lysed with 1% triton X-100 (Biorad, Hercules, CA, USA), and frozen overnight before the fluorescence intensity (385/535 nm) was measured using the Wallac 1420 Victor 3TM Plate Reader (PerkinElmer, Boston, MA, USA). The fluorescence intensity of lysed cells identified bound PBMCs per test sample area.

### 4.5. Statistics

Data were presented as mean ± standard deviation (SD), and analyzed with the Wilcoxon-Signed-Rank-Test (Sigma-Stat, SPSS, Chicago, IL, USA) after passing the Friedman-Test (Sigma-Stat). p-values ≤ 0.05 were considered significant. All analyses in the static cell culture were done with six different cultures in quadruplicate. The amount of experiments in the dynamic setting was specified in the respective data presentation.

## 5. Conclusion

Titaniferous upgrading of commercially available biomedical-grade PUs using PACVD technique is a suitable method to improve cytocompatibility of blood-contacting devices. The thin Ti(C,N,O) layer promotes EC proliferation onto PEU but not onto PCU. Furthermore, the haemocompatibility of titaniferous surfaces is very inconsistent.

## Figures and Tables

**Table 1.** Haemocompatibility of pure and titaniferous upgraded polyurethanes.

Test samples	Product name	*n*	Cooper’s classification [17]	Cell density (cells per μm^2^)	*p*-value	Length of pseudopodia (μm)
PU_1	Texin^®^ 985	4	3–4	>60	–	collagen network
Ti_PU_1	–	4	2	6 ± 6	0.029	2.6 ± 0.9
PU_2	Tecothane^®^	4	2	9 ± 1	–	3.4 ± 1.4
Ti_PU_2	–	4	4	>60	0.057	collagen network
PU_3	Elastollan^®^	5	3	8 ± 8	–	2.2 ± 0.9
Ti_PU_3	–	5	2	13 ± 3	0.421	2.7 ± 0.9
PU_4	Desmopan^®^	4	1*	0.3 ± 0.3	–	0.3 ± 0.4
Ti_PU_4	–	4	1*	0.4 ± 0.4	0.786	no pseudopodia

Ti_PU, titaniferous upgraded polyurethane (PU); Cooper’s classification see Section 4.3

* only a few adherent cells were detectable.

**Table 2.** Overview of used polyurethanes.

Test samples	Type	Product	Manufacturer
PU_1	PEU	Texin^®^ 985	Bayer, Leverkusen, Germany
PU_2	PEU	Tecothane^®^ TT1085	Lubrizol, Wickliffe, OH, USA
PU_3	PEU	Elastollan^®^ 1180A	BASF, Ludwigshafen, Germany
PU_4	PCU	Desmopan^®^	BASF, Ludwigshafen, Germany

PEU, polyether-based polyurethane; PCU, polycarbonate-based polyurethane.
